# Transmural remodeling of right ventricular myocardium in response to pulmonary arterial hypertension

**DOI:** 10.1063/1.5011639

**Published:** 2017-12-12

**Authors:** Reza Avazmohammadi, Michael Hill, Marc Simon, Michael Sacks

**Affiliations:** 1 Willerson Center for Cardiovascular Modeling and Simulation, Institute for Computational Engineering and Sciences, Department of Biomedical Engineering, The University of Texas at Austin, Austin, Texas 78712, USA; 2 School of Mathematical Sciences, University of Nottingham, Nottingham NG7 2RD, United Kingdom; 3 Departments of Cardiology and Bioengineering, Heart and Vascular Institute, University of Pittsburgh, Pittsburgh, Pennsylvania 15260, USA

## Abstract

Pulmonary arterial hypertension (PAH) imposes substantial pressure overload on the right ventricular free wall (RVFW), leading to myofiber hypertrophy and remodeling of its collagen fiber architecture. The transmural nature of these adaptations and their effects on the macroscopic mechanical behavior of the RVFW remain largely unexplored. In the present work, we extended our constitutive model for RVFW myocardium to investigate the transmural mechanical and structural remodeling post-PAH. Recent murine experimental studies provided us with comprehensive histomorphological and biaxial mechanical data for viable, passive myocardium for normal and post hypertensive cases. Multiple fiber-level remodeling events were found to be *localized* in the midwall region (40% < depth < 60%): (i) reorientation and alignment of both myo- and collagen fibers towards longitudinal (apex-to-outflow tract) direction, (ii) substantial increase in the rate of the recruitment of collagen fibers with strain, and (iii) a corresponding increase in the mechanical interactions between the collagen and myofibers. These adaptations suggest a denser and more fibrous connective tissue in the midwall region, and led to a substantially stiffer mechanical response along the longitudinal direction in post-PAH tissues. Moreover, using a Laplace-type mechanical equilibrium analysis of the right ventricle to approximate the wall stress state, we estimated that the longitudinal component of stress remained higher in the hypertensive state while the circumferential component approximately maintained homeostasis values. This result was consistent with our observation from the fiber- and tissue-level remodeling that longitudinally oriented collagen fibers, localized in the midwall region, dominated the remodeling process. The findings of this study highlight the need for more integrated cellular-tissue-organ analysis to better understand the remodeling events during PAH and design interventions.

## I. INTRODUCTION

Right ventricular (RV) failure is a major cause of mortality for patients suffering from pulmonary arterial hypertension (PAH) with a mortality rate of 37.2% at 3 years post-diagnosis.^1,2^ The health and status of the right ventricle of the heart has been shown to be a key indicator of overall progression of PAH,^3^ and thus, a good predictor of survival for patients suffering from this diseases. From mechanistic point of view, PAH imposes a pressure overload on the RV, leading to elevated wall stress, and subsequently to progressive hypertrophy and remodeling. This ultimately results in mechanical failure of the right heart.^4,5^ In recent years, there have been many clinical studies assessing the hypertrophy and remodeling processes taking place during PAH,^6–8^ as well as in investigating whether or not these processes are reversible.^9,10^ The foundation of such studies must rest on an understanding of changes in the structure-function relationship that occur during RV hypertrophy and remodeling. Such an understanding is essential in developing computational biomechanical models^11–13^ of the RV. These models can be used to predict the onset and progression of PAH, and hence, provide means to evaluate potential therapies designed to cure PAH in a more cost-effective and expeditious manner. Yet, relatively little known of the structural and mechanical alterations of the right ventricular free wall (RVFW) myocardium during PAH and its relation to parallel changes in RV function.

**FIG. 1.**
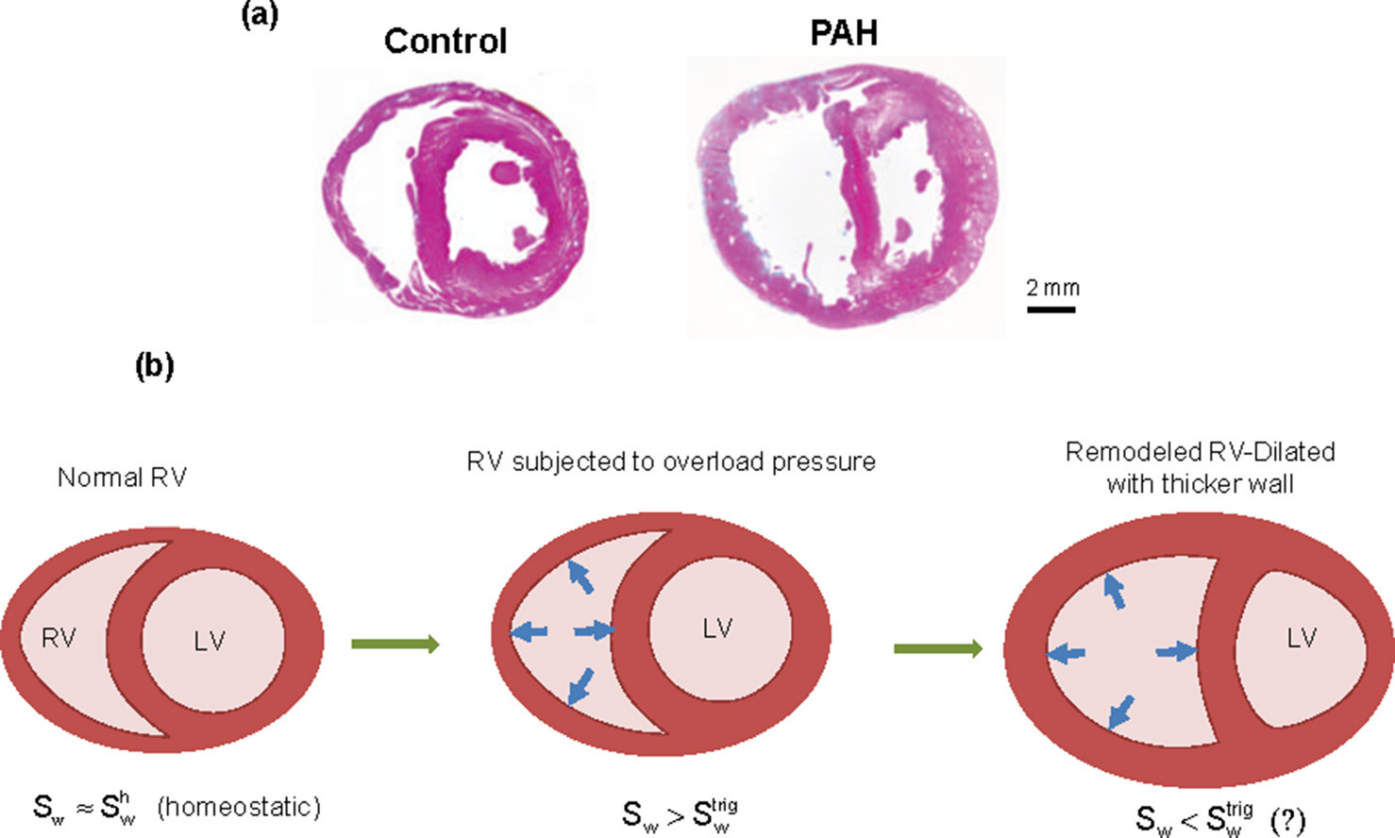
(a) An example of growth in a truncated rat heart under pulmonary hypertension. (left) Normal state, (right) hypertrophic state. [Reproduced with permission from Hirata *et al.*, BioMed Res. Int. **2015**, 1–10 (2015). Copyright 2015 Author(s), licensed under a Creative Commons Attribution 3.0 Unported License.)].^15^ (b) Schematic biventricular configurations in various stages of adaptation to a sustained, overload pressure. (left) Homeostatic state, (middle) hypertrophy-triggered state, (right) RV-remodeled state. S_w_, wall stress; $S_{w}^{h}$, homeostatic value; $S_{w}^{\text{trig}}$, trigger value.

To restore RV cardiac output in PAH to normal values, the right ventricle (RV) undergoes marked growth and remodeling (G&R) at multiple length scales. A detailed knowledge of G&R mechanisms at the fiber level is important to understand mechanical and hemodynamic changes in RV function at tissue and organ levels. The right ventricular free wall (RVFW) consists of myofibers (also known as cardiomyocytes or muscle cells), collagen fibers, a vascular network, and an amorphous ground matrix.^14^ The underlying mechanisms of G&R responses at the fiber level to the increased ventricular pressure can be of two main types. The first type, associated with growth (hypertrophy), induces changes in mass and volume of myo- and collagen fiber aggregates [Fig. 1(a)]. The second type is associated with remodeling, and induces alterations in mechanical and structural properties of myofiber and collagen fiber ensembles.^15^

Details of G&R mechanisms at the fiber level, including the local transmural variation and the contribution of each mechanism on the altered tissue-level behavior, remain largely unknown. Along these lines, we recently developed a novel structurally based constitutive model for the RVFW myocardium that separates the contributions of myofibers and collagen fibers and accounts for their interactions. In addition, the model can be extended to examine the transmural variations in the mechanical contribution of each fiber type. These capabilities allow us to explore and gain new insights into the fiber-level adaptations particular to each fiber type and the transmural locality of these adaptations in response to PAH. Such insights from modeling are essential to both extend and guide experimental studies of the remodeling process. In particular, the interaction contribution included in our model may indicate the level of mechanical engagement of fine collagenous network running across myofibers and large (perimysial) collagen fibers. As the changes in the properties of this network are difficult to measure experimentally, our model can be used to predict changes in the mechanical and architectural properties of the collagenous network in response to pressure overload. In this sense, the applicability of the model in providing new insights into fiber-level adaptations of the RVFW remains to be explored.

The next important step is to understand how the adaptations at the fiber and tissue levels correlate with the changes in the RV's response to PAH at the organ level. In particular, recent studies^14,16^ on the mechanical and morphological properties of normal and hypertensive RVFW myocardium strongly suggest that myocardial wall stress is the primary mediator of RVFW growth and remodeling responses. This is consistent with the traditional view that G&R responses are triggered by a disturbance in the homeostatic equilibrium of local stresses.^17^ Further studies^18,19^ suggested that, among several possible stress and strain stimuli, the Cauchy stress is a reasonable candidate to mediate the remodeling process in cardiovascular tissues. In particular, the quantification of the in-vivo wall stress can help to determine if there exists a threshold wall stress level beyond which RV hypertrophy and remodeling take place due to pressure overload to restore the wall stress values [see Fig. 1(b)]. Moreover, studying the changes in the wall stress from normal to post-PAH can provide insights into determining if certain G&R mechanisms are adaptive or maladaptive.

Our objective in the present work was to utilize our novel myocardium constitutive modelling framework^20^ to investigate RV remodeling on the passive behavior of the hypertension-induced, non-contracting, viable RVFW myocardium. To estimate the parameters of the model, we used the extensive mechanical and transmural histological data from recent murine experimental studies for both control (normal) and post (3-week) hypertensive states.^14,16^ In contrast to prior works^14,16^ that provided insights into the “global” remodeling mechanisms during PAH, the current study aimed to quantify and analyze changes in the contribution of each fiber type at the tissue level and the transmural “locality” of these changes, using the implementation of the fiber-specific structural model. Moreover, using a Laplace model, we investigated the correlation between fiber-level adaptations and the changes in the wall stress components at the organ level. Comparison of parameters of the model estimated for the control and 3-week hypertensive specimens suggested new and quantitative insights into the “localized” microstructural remodeling of the RVFW in response to the development of pulmonary hypertension.

## II. RESULTS

The biaxial mechanical behavior data of hypertensive RVFW specimens were well fit by the constitutive model (3), with $r^{2}>0.95$ and $MSR=\sqrt{\Sigma (\delta )/N}<1.1\;(kPa)$ calculated at the optimal values. Estimated values of all unknown parameters in the model are presented in Table I.

**TABLE I.** List of estimated model parameters (n = 3).

Ground matrix	(Perymysial) collagen fibers						
$k^{g}$_ _(kPa)		Recruitment			Transmural variation		
	$k^{c}$ (MPa)	$E_{lb}$	$\mu_{r}$	$\sigma_{r}$	$E_{ub}^{m}$	$k_{z}^{c}$	$z^{c}$
10	7.02 ± 0.3	0.015 ± 0.006	0.067 ± 0.01	0.0027 ± 0.002	0.072 ± 0.009	2.14 ± 0.9	54.15 ± 8.9

Myofibers		Myo-collagen fiber interaction			
			Transmural variation		
$k_{1}^{m}$ (kPa)	$k_{2}^{m}$	$k_{2}^{mc}$	$k_{1m}^{mc}$ (kPa)	$k_{z}^{mc}$	$z^{mc}$
58.07 ± 1.6	1.38 ± 1.7	8.42 ± 2.1	0.51 ± 0.3	5.11 ± 3.4	55.5 ± 9.4

### A. Histology adaptation to overload

**FIG. 2.**
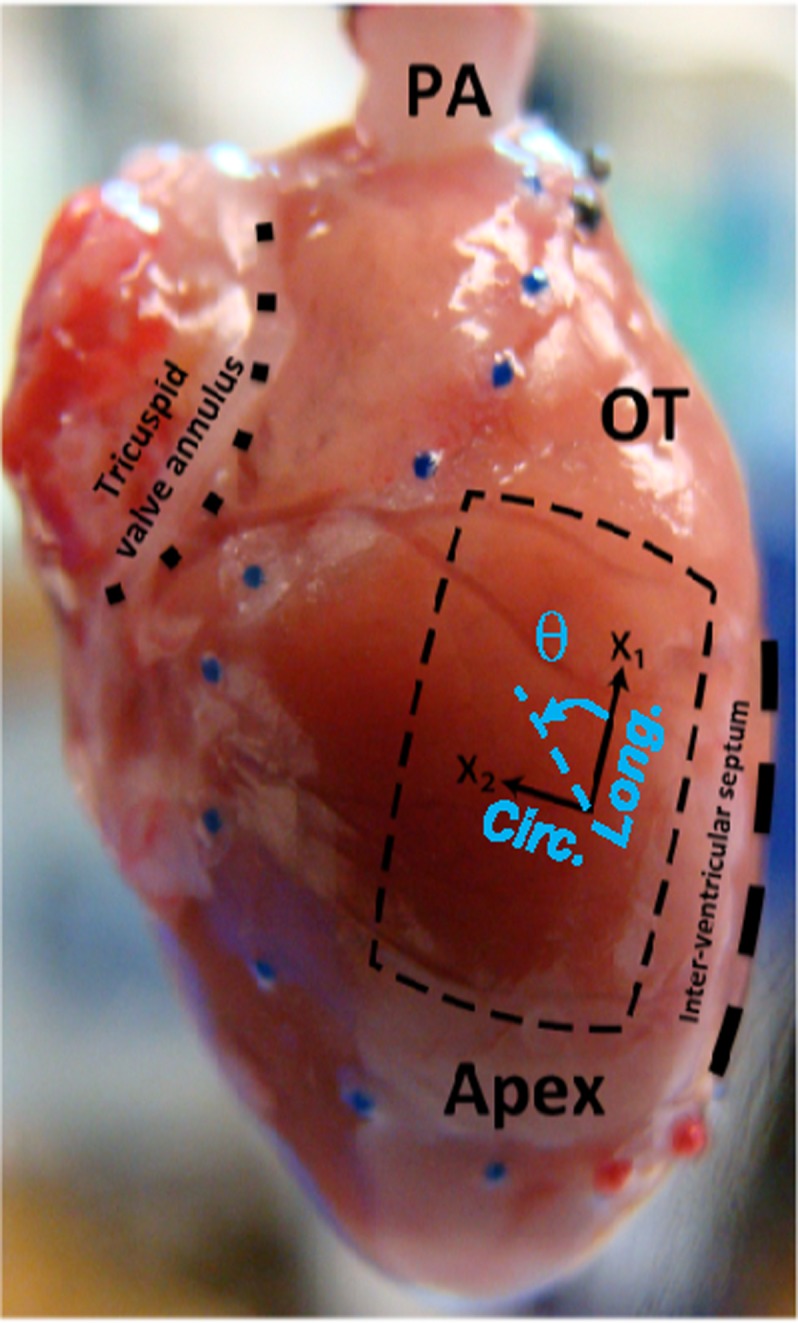
Isolated rat heart and right ventricular free wall (RVFW), denoted by a square slab (crossed lines), and the coordinate basis {x_1_,x_2_} used for histological measurements. The directions x_1_ and x_2_ approximately represent the longitudinal (apex-to-outflow tract) and circumferential directions, respectively. Note that the transmural direction is perpendicular to the x_1_-x_2_ plane. The angle θ (positive anticlockwise) denotes the orientation of myo- and collagen fibers (denoted by θ^m^ and θ^c^, respectively) in the x_1_-x_2_ plane. Reproduced with modification with permission from Valdez-Jasso *et al*., J. Physiol. **590**, 4571-4584 (2012). Copyright 2012 John Wiley and Sons.

**FIG. 3.**
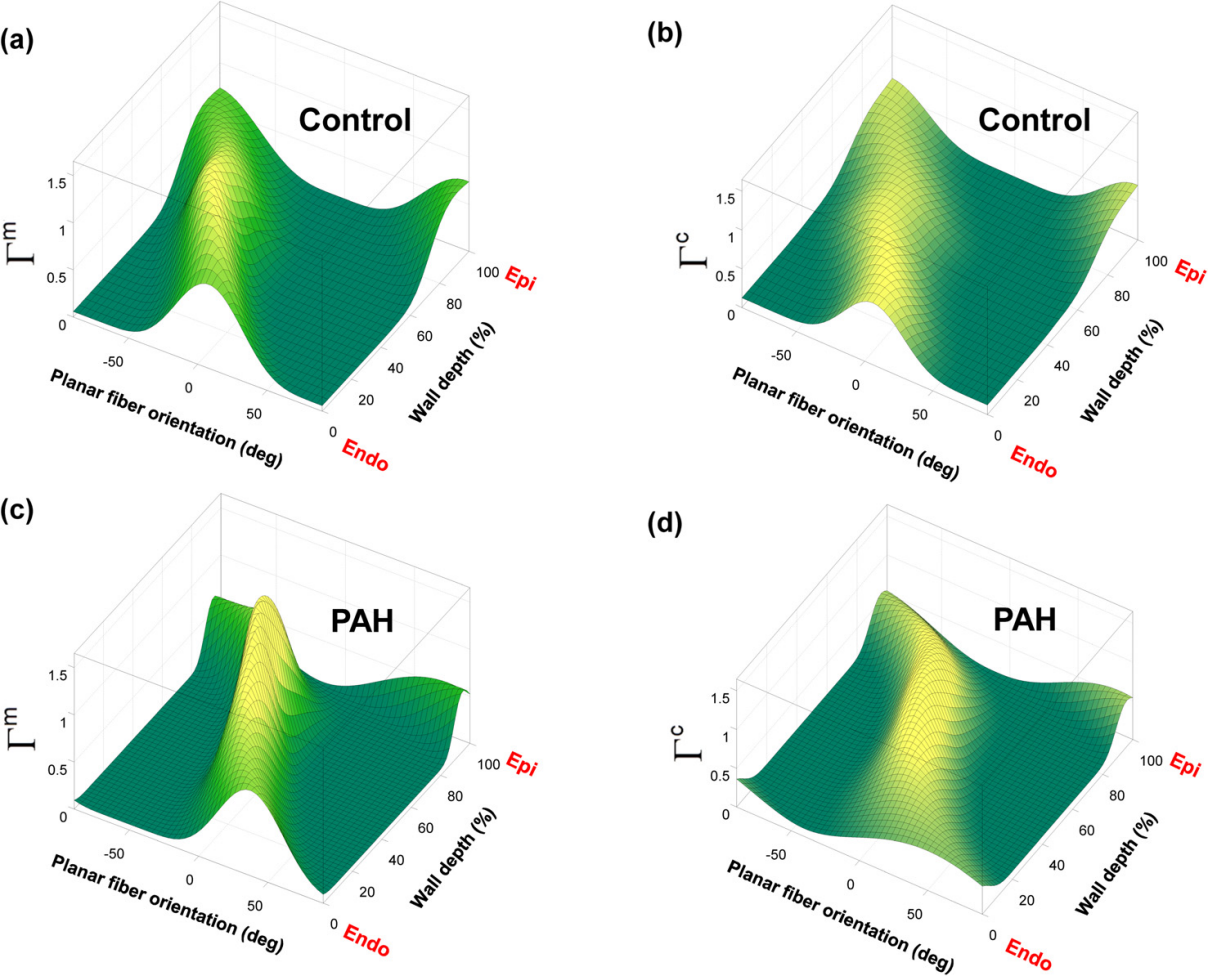
3D Beta-distribution surface fit to transmural measurements of fiber orientation distribution in RVFW. [(a), (b)] Orientation distribution of myo- and (perimysial) collagen fibers for the normal specimens. [(c), (d)] Orientation distribution of myo- and (perimysial) collagen fibers for the post-PAH specimens.

**FIG. 4.**
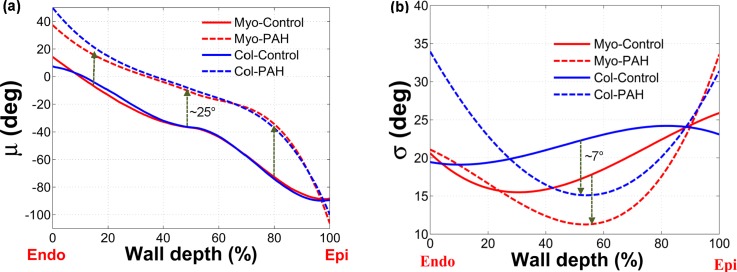
Statistical measurements of the transmural orientation distribution for myo- and (perimysial) collagen fibers for normal and post-PAH specimens as functions of the wall depth. (a) Mean. (b) Standard deviation.

The 3D Beta-distribution surfaces extracted from histological analysis of 3-week PAH tissues revealed that both myo- and collagen fibers exhibited a fairly uniform (full-thickness) shift in the orientation towards the longitudinal direction (i.e., apex-to-base direction aligned with θ=0°, see Fig. 2) with a more pronounced alignment of fibers in the midwall region, again towards the longitudinal direction (Fig. 3). Also, myo- and collagen fibers showed a very close orientation at all depths, similar to the control (normal) case (Fig. 3). Mean fiber orientation (μ) of both myo- and collagen fibers in the post-PAH tissue showed a stronger deviation from a linear behavior compared to the control case [Fig. 4(a)], especially for the region closer to the epicardial surface (*z* = *H*). The standard deviation of fiber orientation (σ) for both myo- and collagen fibers was significantly smaller in the midwall region (40≤z≤60) indicating higher alignment of fibers towards longitudinal direction in this region [Fig. 4(b)].

### B. Biaxial mechanical behavior

**FIG. 5.**
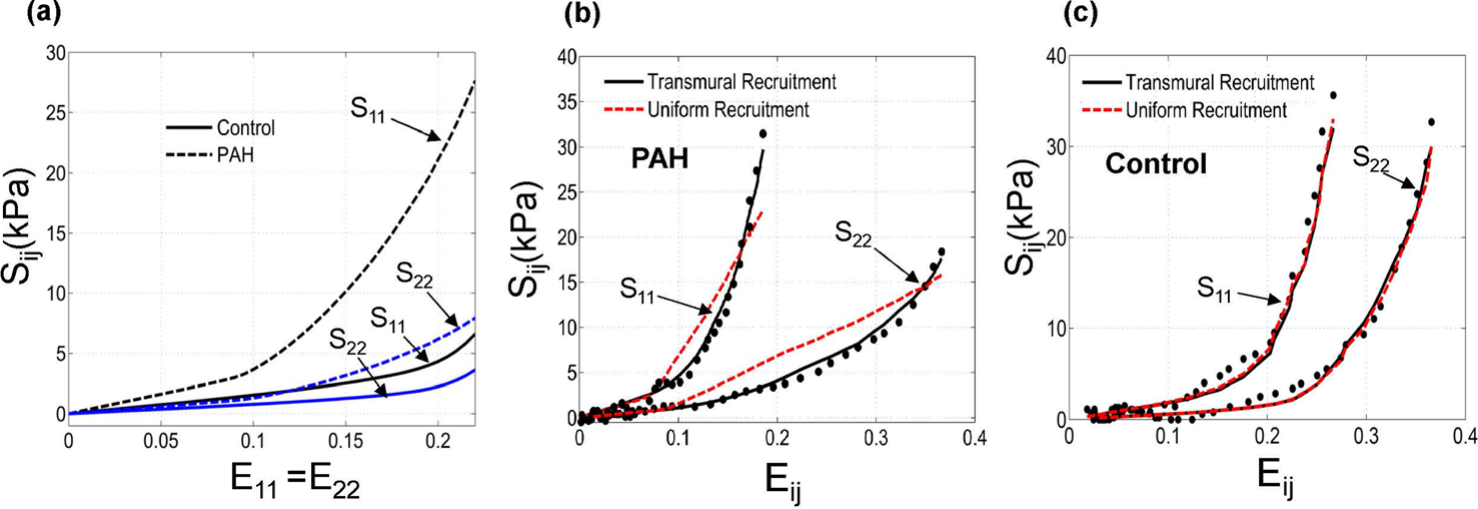
(a) Prediction of our model for the overall mechanical response of the RVFW tissue under equibiaxial strain path (E_11_ = E_22_) for control and PAH cases. (b) A representative fit of our constitutive model (solid lines) to the PAH tissue response (markers). The corresponding results of the “uniform-recruitment” model are included for comparison (dashed lines) where the upper bound strain (E_ub_) and the interaction module ($k_{1}^{mc}$) are constant within the depth. The parameters in each model were separately estimated. The horizontal axis represents E_11_ and E_22_ for S_11_ and S_22_, respectively. (c) The corresponding fit for the case of a control specimen. The result indicated that a transmural variation of recruitment properties was necessary to capture the mechanical behavior of PAH specimens.

As discussed in detail in previous works,^14,16^ the overall mechanical anisotropy of PAH tissues significantly increased compared to the control case [Fig. 5(a)]; the ratio of stress in mean fiber direction over that in cross-fiber direction multiplied by ∼2. Also, PAH tissues showed a large decrease in the extent of the “toe region” (the strain range within which collagen fibers are recruited) leading to a pronounced stiffening in the tissue response [Fig. 5(a)].

### C. Fiber contributions

**FIG. 6.**
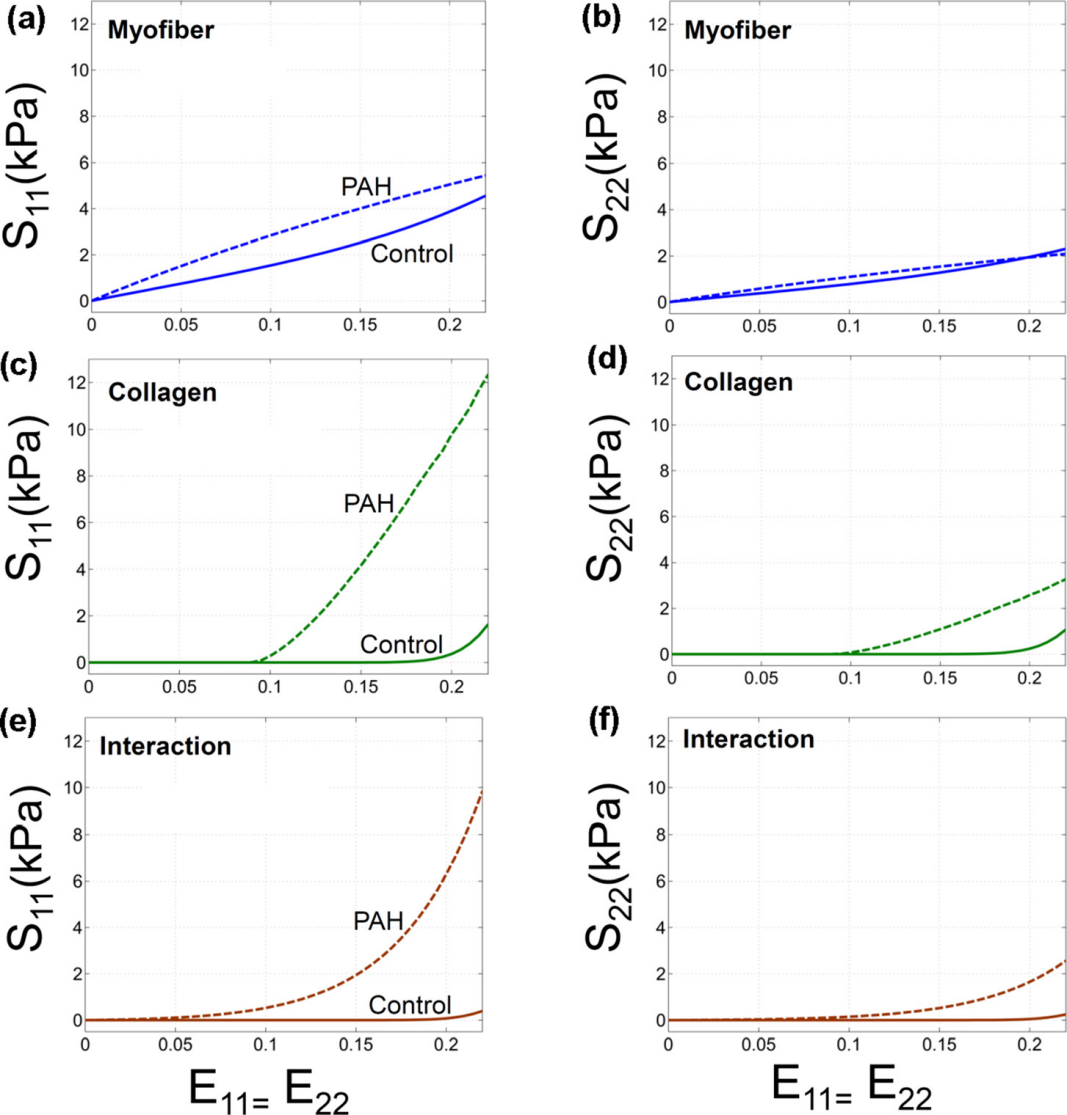
Prediction of our model for contributions of myo- and collagen fibers and interaction among them in the stress response as function of the applied strain under equibiaxial strain path (E_11_ = E_22_). The corresponding contributions for the case of control are included for comparison. [(a), (b)] Myofiber, [(c), (d)] Collagen, [(e), (f)] Interaction.

The qualitative contribution of myofibers and (perymysial) collagen fibers remained unchanged in the PAH tissues such that the behavior of the RVFW was still governed by myofiber response alone in the low strain regime,^33^ and collagen fibers and their interaction with myofibers began to gradually contribute to the behavior as their recruitment proceeded (Fig. 6). However, in contrast to the control case, the collagen fibers were recruited within a significantly smaller range of the applied strain and the mechanical interaction between myo- and collagen fibers tended to be stronger (Fig. 6, the upper bound strain, $E_{ub}^{m}$, decreased from 0.21±0.015 to 0.072±0.009). Similar to the control case, the contribution of the interaction was found to be the highest under equibiaxial strain path.

### D. Transmural variations of recruitment and wall stress

**FIG. 7.**
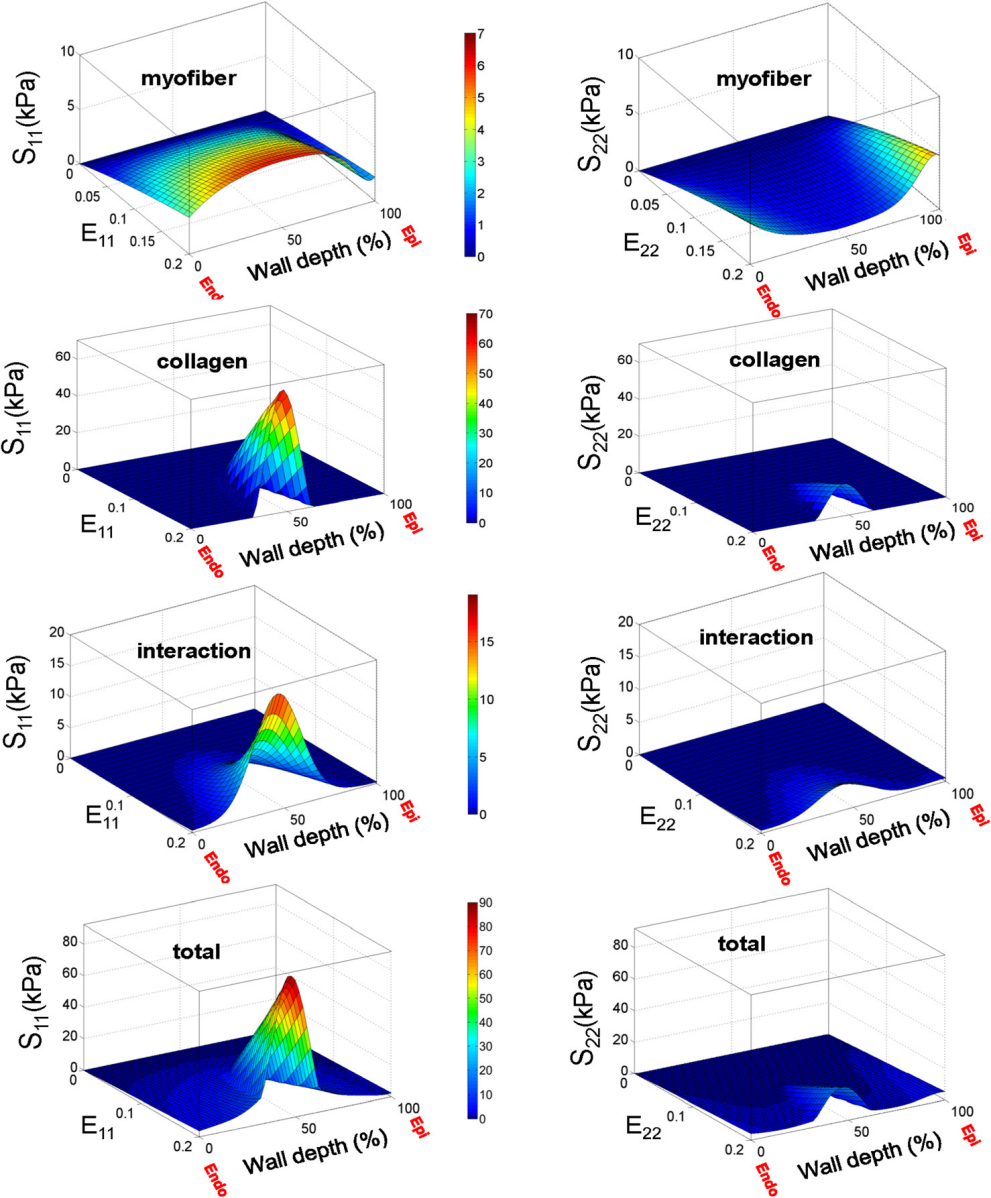
Prediction of our model for contributions of myofibers, collagen fibers and their interaction in the total stress for PAH specimens under equibiaxial strain path E_11_ = E_22_.

The transmural variation in the recruitment properties and correspondingly in the interaction strength was necessary in our model to consistently capture the mechanical behavior of the PAH tissues under various loading conditions [see Fig. 5(b) for a representative protocol], although this was not the case for normal RVFW specimens [see Fig. 5(c) for a representative protocol]. Stress analysis of the PAH biomechanical data predicted substantial transmural variations in the fiber stress (Fig. 7). The mechanical contribution of collagen fibers showed the strongest transmural variation, while the variations in the contribution of myofiber were small (Fig. 7). Also, as expected, the contribution of fiber-fiber interaction exhibited a weaker variation than that of collagen fibers influenced by the nearly uniform contribution of myofibers. The transmural variations were generally stronger in the component *S*_11_ corresponding to the stress in the longitudinal direction.

**FIG. 8.**
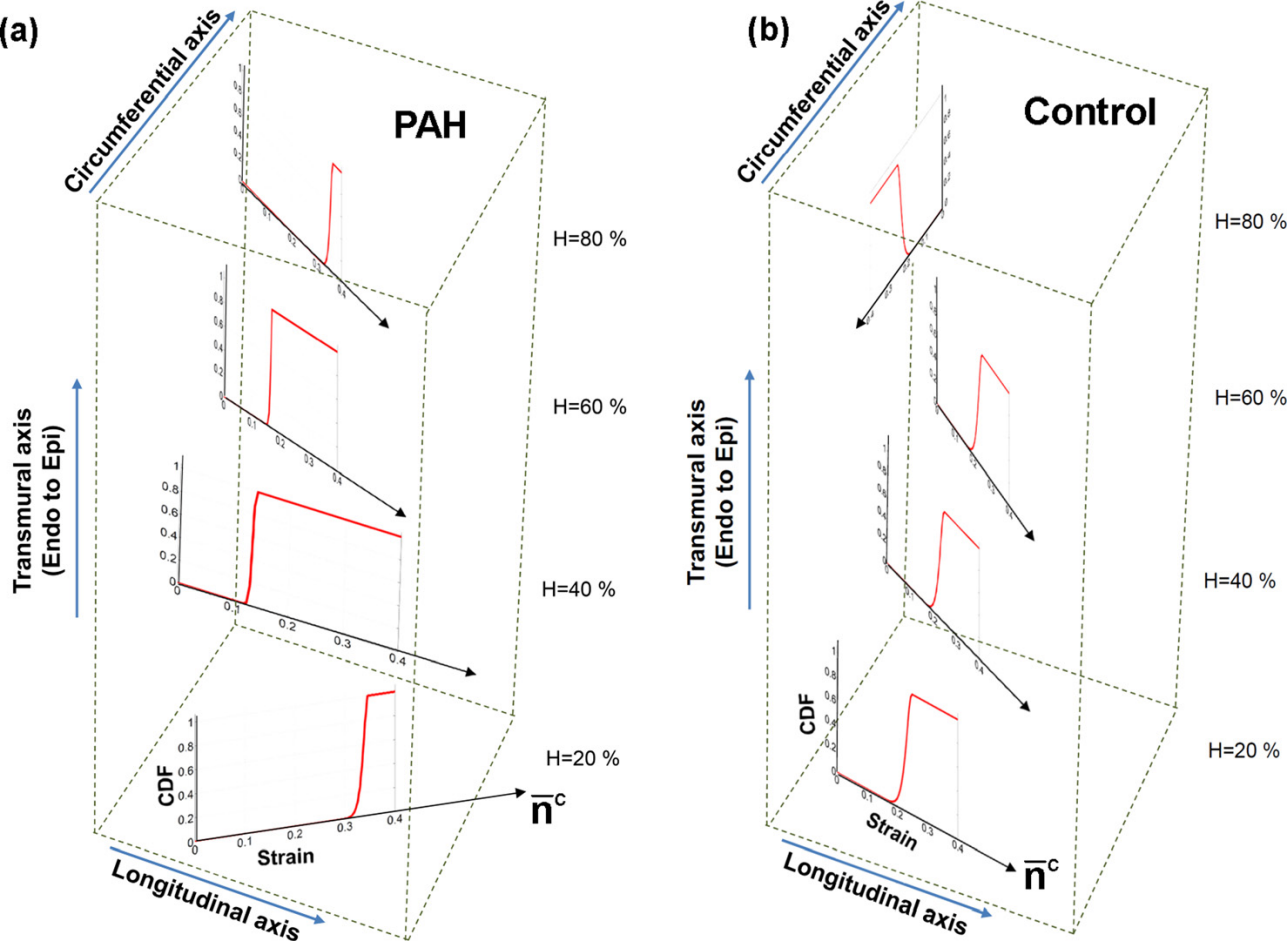
Predictions of our model for the transmural distribution of collagen fiber recruitment under equibiaxial strain path (E_11_ = E_22_). The plots show the cumulative distribution function (CDF) of the recruitment function D. (a) PAH, (b) Control. The plots are stacked to represent the respective wall depth and the transmural variation in the average orientation of collagen fibers (denoted by ${\bar{n}}^{c}$).

**FIG. 9.**
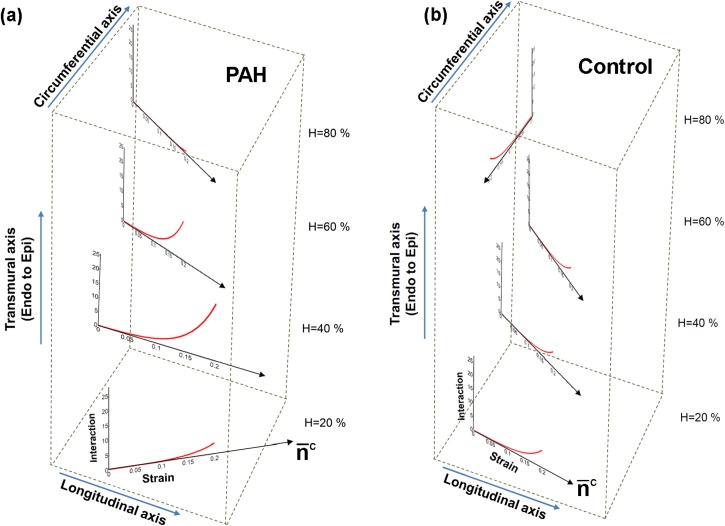
Predictions of our model for the transmural contribution of the fiber-fiber interaction in the stress ($S_{ens}^{m-c}=S_{11}^{m-c}+S_{22}^{m-c}$) under equibiaxial strain path (E_11_ = E_22_). (a) PAH, (b) Control. The plots are stacked to represent the respective wall depth and the transmural variation in the average orientation of collagen fibers (denoted by ${\bar{n}}^{c}$).

The variation in the stress contribution of collagen fibers stemmed from the strong variation in the fiber recruitment process along the wall thickness (Fig. 8). The results for the cumulative distribution function (CFD) of the recruitment function [$D(\lambda_{s},z)$] indicated that collagen fibers are recruited markedly sooner with strain (corresponding to smaller $E_{ub}$) in the midwall region while some fibers closer to epi- and endocardial surfaces did not reach full recruitment within the physiological strain range [Fig. 8(a)]. In contrast, the constitutive model predicted a uniform recruitment across the RVFW thickness for the case of normal specimens [Fig. 8(b)]. Correspondingly, (perimysial) collagen fiber stress and the interaction with myofibers were pronounced in the midwall region (40≤z≤60) and diminished beyond this region [Fig. 9(a)]. Such variation was not predicted for the case of normal specimens [Fig. 9(b)].

### E. Akaike Information Criterion (AIC) values and parameter correlations

The AIC values calculated before and after incorporation of the parameters $\kappa =\{k_{z}^{c},z^{c},k_{z}^{mc},z^{mc}\}$ in the model indicated that enabling model to account for the transmural variations in the recruitment and fiber interaction significantly improved the fit quality for the post-PAH tissues, while did not justify the inclusion of these parameters for the normal tissues (Table II). The sensitivity matrix columns were calculated by varying each of the parameters in a range of ±0.01% around their estimated values, while the remaining parameters were kept constant [see Table III for correlation coefficients between the selected parameters $\kappa =\{k^{c},E_{ub}^{m},k_{z}^{c},z^{c},k_{1}^{mc},k_{z}^{mc},z^{mc}\}$]. The closer the absolute value of $CR_{\mathrm{ij}}$ is to 1, the higher is the correlation between the parameters $\kappa_{i}$ and $\kappa_{j}$. The calculated coefficients indicated low to moderate correlations for most of the parameters suggesting a fairly strong identifiability of the model.

**TABLE II.** AIC values before and after incorporating the transmural variations of recruitment and interaction in the model.

	Control	PAH
Without transmural variation	196.24	289.46
With transmural variation	204.44	74.98

**TABLE III.** Correlation coefficients between selected parameters.

	$k^{c}$	$E_{ub}^{m}$	$k_{z}^{c}$	$z^{c}$	$k_{1}^{mc}$	$k_{z}^{mc}$	$z^{mc}$
$k_{1}^{m}$	1	−0.15	−0.48	−0.24	−0.53	0.49	−0.65
$E_{ub}^{m}$		1	0.42	−0.08	−0.15	−0.09	0.32
$k_{z}^{c}$			1	0.01	0.16	−0.18	0.48
$z^{c}$				1	0.45	−0.15	0.20
$k_{1}^{mc}$					1	−0.48	0.44
$k_{z}^{mc}$						1	−0.83
$z^{mc}$							1

### F. Changes in the average wall stress

**FIG. 10.**
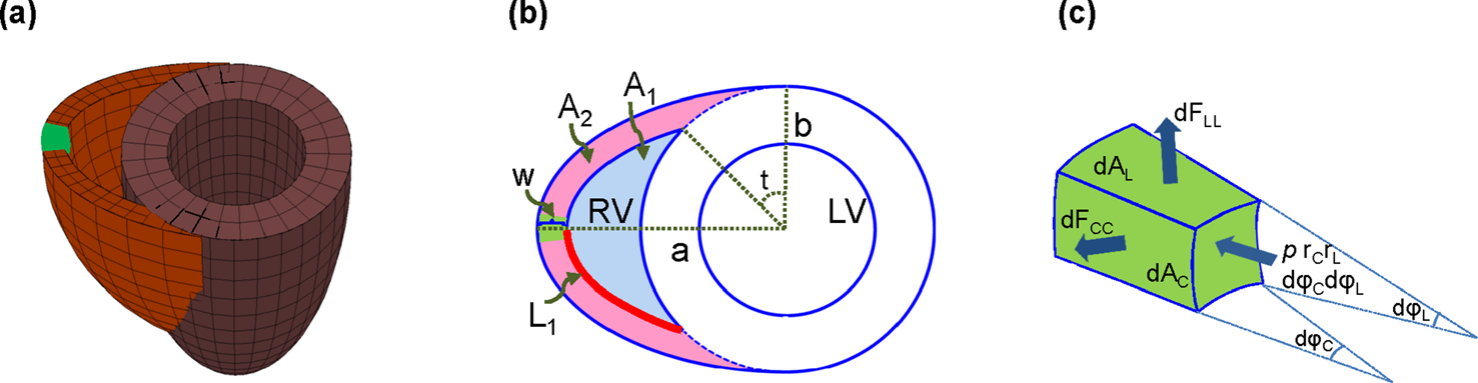
(a) Simple biventricular model used to estimate the average wall stress in the green element. (b) Top view of the biventricular model with relevant geometrical parameters. (c) The cut element from the model showing the longitudinal and circumferential force components and the applied pressure.

The Laplace model predicted that the value of the circumferential stress component ($\sigma_{\text{CC}}$) was larger than that of the longitudinal component ($\sigma_{\text{LL}}$) in both control and post-PAH cases (Table IV). This is similar to the results for the left ventricular (LV) wall stress^30^ using a simple prolate spheroidal geometry. Our analysis further predicted that, after sustaining a nearly tripled pressure for three weeks, the circumferential stress was approximately restored to the normal value while the longitudinal component remained higher than the corresponding normal value (Table IV). More specifically, the hypertensive values of $\sigma_{\text{CC}}$ and $\sigma_{\text{LL}}$ were reduced by 84% and 33%, respectively, from the corresponding values calculated for only the increase in the pressure and no geometrical changes. Our calculations indicated that, in addition to the increase in the wall thickness, the RV was enlarged [corresponding to a larger *a* in Fig. 10(b)] after three weeks of sustaining the pressure overload (Table IV). This increase in *a*, according to Eqs. (20)_3–4_, leads to an increase in radii $r_{L}$ and $R_{L}$ implying that the oblate spheroid becomes more flat at the green element in Fig. 10(a) along the longitudinal axis. This change of shape is consistent with the sphericalization of the RV observed in PAH hearts.^14,16^

**TABLE IV.** Approximate values for RV wall stress and biventricular dimensions (n = 5) with reference to Fig. 10.

	p (kPa)	$V\;\left({\mathrm{mm}}^{3}\right)$	a (mm)	b (mm)	w (mm)
Normal	3.73 ± 0.16	75 ± 52	7.98 ± 0.07	4.61 ± 0.2	0.75 ± 0.01
Hypertensive	12.6 ± 0.75	160 ± 119	10.91 ± 0.16	4.84 ± 0.4	1.23 ± 0.05

	$\sigma_{CC}\;\left(kPa\right)$	$\sigma_{LL}\;\left(kPa\right)$
Normal	8. 56 ± 1.05	4.16 ± 0.6
Hypertensive	11.85 ± 1.65	10.60 ± 1.1

## III. DISCUSSION

### A. Overall findings

**FIG. 11.**
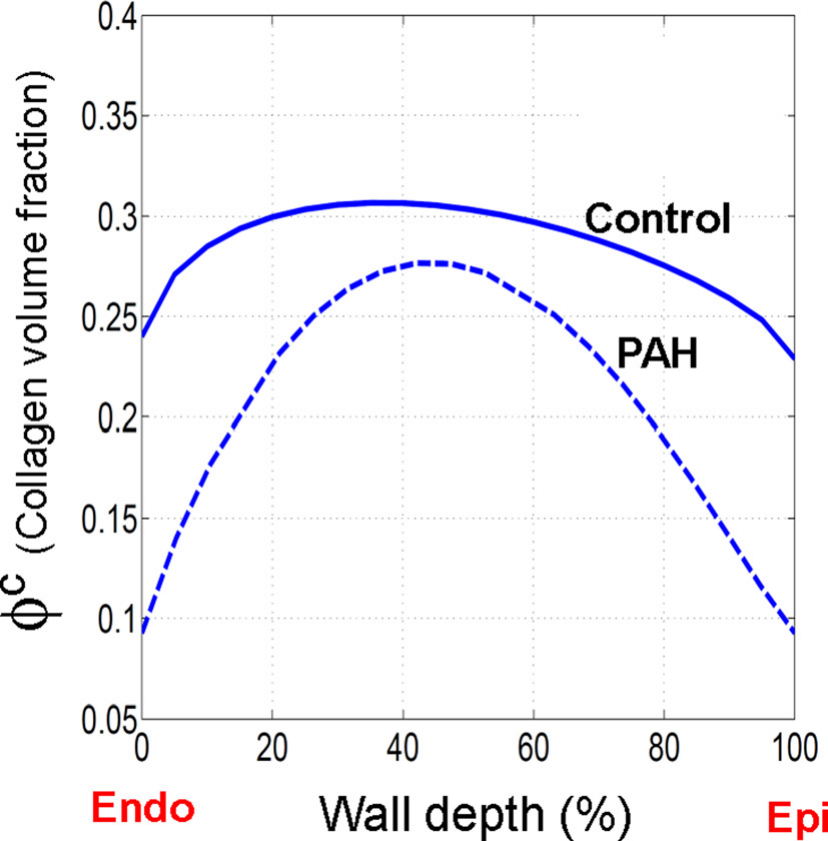
Transmural distribution of the collagen fiber volume fraction. The lines represents Beta distribution fits to the experimental measurements of the volume fraction.

In this work, we took an essential step towards the quantification and simulation of the spatial *locality* of G&R drivers underlying the development of PAH. Specifically, to identify and quantify possible transmural adaptations of mechanical properties of the RVFW under PAH, we applied our structural model (differentiating between mechanical contributions of myo- and collagen fibers) and informed it with highly detailed, transmural histological data for myo- and collagen fibers (Fig. 3). All PAH specimens exhibited an augmented anisotropic stress-strain behavior with strong stiffening along the longitudinal (apex-to-the outflow tract) direction [Fig. 5(a)]. The stiffening in this direction was found to be the direct result of several fiber-level adaptations “localized” in the midwall region:

• The volume fraction of collagen fibers was significantly higher in the midwall region (Fig. 11)
• The (perymysial) collagen fibers and myofibers underwent a large collective reorientation towards the longitudinal direction [Fig. 4(a)] and significant alignment in the midwall region along this direction [Fig. 4(b)].
• The collagen fibers exhibited both an earlier and faster recruitment with strain in the midwall region [Figs. 6(c), 6(d), and 8] which led to a strong transmural variation in the contribution of collagen fibers in the total stress (Fig. 7). In contrast, the transmural variation of the myofiber contribution to the total stress was small (Fig. 7).
• The mechanical interaction between collagen and myofibers substantially increased in the midwall region [Figs. 6(e), 6(f), 7, and 9].

These findings suggest that, in response to PAH, the connective tissue in the RVFW becomes more dense and fibrous in the midwall region to accommodate most of mechanical loading during the passive inflation. Finally, consistent with both fiber- and tissue-level adaptations, we found from the organ-level analysis (using the Laplace-type model) that the value of the longitudinal stress component remains noticeably higher than the corresponding homeostatic value (Table IV), while the circumferential stress value was almost maintained within the homeostatic range.

### B. Transmural adaptations of the RVFW to RV hypertension

Histological analyses (Fig. 3) revealed substantial localized adaptations of structural properties of myo and collagen fibers in the post-PAH tissue with a prominent alignment in the midwall region [Fig. 4(b)]. The experimental observation of pronounced transmural variation in the volume fraction of collagen fibers (Fig. 11) motivated the need to investigate the transmural adaptations in the effective mechanical properties of collagen fibers and their interaction with myofibers. The AIC values calculated for our model before and after incorporation of the parameters $\kappa =\{k_{z}^{c},z^{c},k_{z}^{mc},z^{mc}\}$ (Table II) supported the hypothesis of significant transmural variations in the rate of recruitment of collagen fiber with strain and the amount of fiber-fiber interactions. Both properties were found to be markedly localized in the midwall region similar to the fiber alignment. Overall, our results indicated that the post-PAH tissues experienced notable transmural stress variations, with the midwall region disproportionately accommodating most of the applied mechanical load (Fig. 7).

### C. Mechanical adaptations of myofibers and collagen fibers

Our parameter estimation study in low strain regime revealed that myofibers exhibited significant stiffening along the longitudinal direction in the post-PAH tissues. (See the longitudinal component of the stress, $S_{11}$, in Figs. 5 and 6 and compare the low-strain regime responses; note that the longitudinal direction is nearly aligned with the mean myofiber direction.) Comparisons of the measured volume fraction and estimated values of myofiber stiffness (Table I) with corresponding values for normal tissues^20^ further indicated that the myofiber stiffening stems primarily from an increase in the stiffness of myofibers (increased by ∼75%), and secondarily from an increase in volume fraction of these fibers (increased by ∼16%). In this connection, we recall from our experimental study^14^ that the myofiber mass increased more than its volume leading to an ∼40% increase to myofiber density. This may explain the increase in myofiber stiffness caused by addition of sarcomeres within the individual cells. It is also interesting to note that the behavior of myofibers was nearly linear for the entire range of strain over which the PAH tissues were tested (the parameter $k_{2}^{m}$ was estimated to be small for most of specimens; see Table I).

**FIG. 12.**
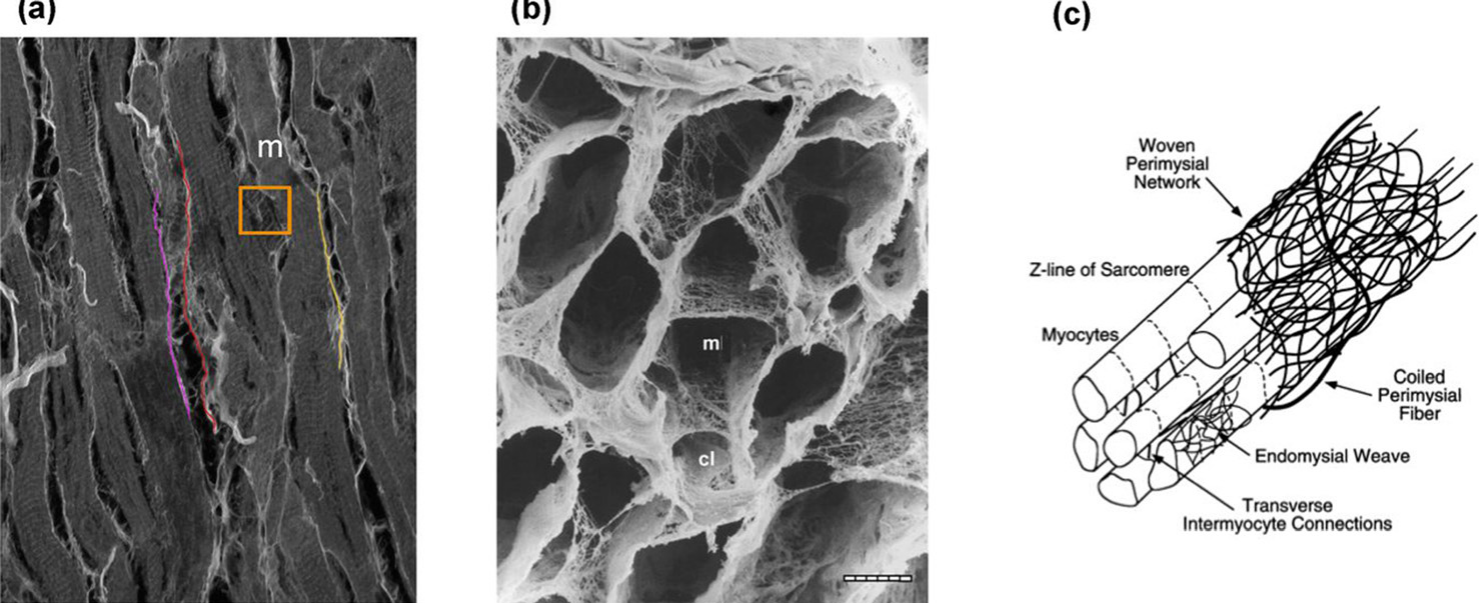
[(a), (b)] Microscopic images of myocardium. (a) Several myofibers (m) and large collagen fibers (colored strands). Note the presence of a dense network of fine collagen fibers (within the orange box). Confocal image. (b) Mesh-like arrangement of fine collagen fiber network (m, myfiber lacunae; cl, capillary lacunae). SEM. Bar = 10 mm. [Reproduced with permission from Macchiarelli *et al.*, Histol. Histopathol. **17**(3), 699–706 (2002). Copyright 2002 Histology and Histopathology.^21^) (c) Schematic representation of cardiac tissue structure showing the arrangement of endomysial and perymysial collagen fibers with respect to myofibers. (From Peterson and Bronzino, *Biomechanics: Principles and Applications*. Copyright 2007 CRC Press. Reproduced with permission from CRC Press.^22^)

Moreover, we found that the interaction between myofibers and collagen fibers substantially increased in the post-PAH tissues, especially along the longitudinal direction (Fig. 6). Recalling that the presence of the “fine” collagen network [Fig. 12(b)] was hypothesized to be a driver of the myofiber-collagen fiber mechanical coupling, this finding suggest additional structural remodeling mechanisms at a smaller scale, such as re-organization of the “fine” collagen network that induces greater myofiber-collagen fiber interaction. Such mechanisms are likely interlinked with the alignment of myo and collagen fibers towards the longitudinal direction [Fig. 4(b)] which overall generates a stronger (extensional) interaction in this direction. Finally, concerning the adaptation of the active behavior of the RVFW to PAH, we expect to see a higher contractile force in the longitudinal direction compared to the control following the realignment of myofibers towards this direction. However, the full quantification and understanding of the changes in the active behavior of the RVFW requires a study of this behavior with the help of a model which has the ability to capture underlying growth and remodeling mechanisms during PAH. The present study on the adaptations of the passive behavior provides an essential platform to investigate changes in the active response in the future.

### D. Wall stress-driven remodeling of the RVFW

According to the Laplace model approximation [Eqs. (18) and (19)], an increase in the RV pressure causes both stress components to increase by the same ratio (as the pressure does) when all geometrical parameters are kept constant. Our measurements^14^ indicated an almost three fold increase in the (end-systolic) pressure from control to PAH (Table IV). However, an increase in the wall thickness and other dimensions nearly restored the circumferential stress although it failed to completely restore the longitudinal stress (Table IV). Based on our formulation for stress calculation in Subsec. V E, this may be because that the RV wall thickening could not keep up with the RV cavity enlargement leading to a higher longitudinal stress. This finding is supported by our observation from histological study of the PAH tissues that both myo- and collagen fibers exhibited a higher alignment in the midwall towards the longitudinal direction, so that they can accommodate elevated deformation caused by the non-restoring longitudinal stress. Also, this alteration in the longitudinal stress appears to have a key role in hastening the recruitment of collagen fibers with strain in the midwall region (where the mean fiber orientation is aligned with the longitudinal direction).

### E. Limitations

The reported transmural variation in the recruitment function was for given measurements of the transmural fiber orientation and volume fraction of fibers. Although imperfections in histology measurements, the Gomori-stained technique, and the subsequent image processing may have influenced the quantitative values of these results, the qualitative trend of faster recruitment of collagen fibers in the midwall region was consistently observed for all of the specimens. Also, the experimental uncertainty in identifying longitudinal direction (apex-to-outflow tract) in each specimen was expected to be small. One limitation with the histological study was that at the given image resolution, the endomysial collagen fibers that surround individual myofibers were not discernable, so that our experimental measurements for collagen fibers predominantly represent perimysial fibers. Another limitation in our work was that the histology measurements (which involved sectioning) and mechanical testing were performed on different specimens. To minimize the error generated by these limitations, a more advanced imaging technique, such as diffusion-tensor magnetic resonance imaging, will be employed in future. Such non-destructive imaging technique will enable us to rapidly quantify myofiber orientation distribution in isolated, perfused, arrested hearts^32^ which allows us to conduct the histology measurement and mechanical testing on the same specimen.^34^

Finally, concerning the Laplace-type model used in this work, it is important to emphasize that we used this idealization only to provide preliminary predictions on the qualitative alterations of average wall stress due to PAH. Such predictions were also limited to only two time points: normal and 3-week hypertensive states. Nonetheless, the predictions were consistent with the tissue longitudinally dominated remodeling. In future, we certainly will need an image-based biventricular finite-element model to fully quantify the stress distribution in the wall at several time points and gain a detailed understanding of stress-driven growth and remodeling process in the RV. The development and verification of this model is in progress.

## IV. CONCLUSION AND FUTURE DIRECTIONS

Utilizing a structure-based constitutive modeling framework, we investigated and quantified the fiber-, tissue- and organ-level adaptations in the RV and the connection between them for fully developed PAH murine specimens. Our modeling approach allowed separation of the tissue-level effects of the mechanical and structural adaptations of myo- and collagen fibers and the interaction between them. We found a significant transmural variation in adaptations of fiber orientation distribution and recruitment. Both myo- and collagen fibers showed a stronger alignment in the midwall region, and collagen fibers adopted a faster recruitment in this region as well. Making connection with the organ-level adaptation, the non-restoring longitudinal stress, predicted by the Laplace model, explained longitudinally dominated remodeling process in the midwall and at the fiber level.

The insights provided in this study lay the foundation for developing a structure-based growth and remodeling model for the RVFW that establishes the link between the driver mechanisms in all three scales of fiber, tissue, and organ. Such a model will provide means to investigate the important question of existence of a “no return” point along the hypertrophy and remodeling progression beyond which the adaptive mechanisms fail to restore the wall stress value. Ultimately, further development and implementation of our model in patient-specific organ-level simulations will allow investigation of optimal diagnosis, new individualized interventions and treatment protocols for PAH.

## V. METHODS

### A. Mechanical testing and structural quantification

Details of the mechanical testing methods and histological analyses have been presented previously.^14^ No animal ethics approval was required for the present study, and the IACUC approval information for the extant data is included in Ref. 14. Briefly, a pulmonary artery (PA) banding model was utilized to create a pulmonary hypertensive condition in rats at the University of Pittsburgh (see Fig. 1 in Ref. 14). A total of 6 hypertensive RVFW specimens of male rats were acquired at 3 weeks post-PAH. Terminal invasive catheter was used to measure complete pressure–volume loop in the RV of control and hypertensive rats (see Fig. 2 in Ref. 14) from which relevant hemodynamic parameters (including end systolic pressure and volume) were derived. Preconditioning was performed, followed by multi-protocol displacement-controlled biaxial testing that encompasses the estimated physiological strain range.

The orientation distribution as well as the transmural volume fractions of myofibers and collagen fibers were determined by analyzing histologically stained sections (layers) of specimens (n = 3). Details of the staining techniques and image post processing have been presented previously.^14^ Briefly, 5-*μ*m thick adjacent sections of myocardium were stained with Gomori One-Step Trichrome, chosen so that myofibers appear pink and collagen gray. The sections were imaged at the resolution of 6.35 *μ*m/pixel. A thresholding algorithm was developed to separate the pink and gray pixels [Fig. 3(a) in Ref. 14]. The pink pixels were analyzed to compute the myofiber orientation distribution as well as the myofiber content per 5-*μ*m section. The pink pixels were removed from the image, and the gray pixels were used to compute the corresponding data for collagen fibers via a similar procedure. Volume fractions of myofibers and collagen fibers, denoted by $\phi^{m}(z)$ and $\phi^{c}(z)$, respectively, were computed assuming the area of each constituent to be uniform over the section thickness. Also, the volume fraction of the amorphous matrix ($\phi^{g}$) was calculated by counting the pixels that were neither pink nor dark gray. This fraction was estimated to be about 3% for both normal and PAH cases ($\phi^{g}=0.03$). Next, following the recent approach,^20^ we used 3-D Beta distribution functions $\Gamma^{m}(\theta^{m},z)$ and $\Gamma^{c}(\theta^{c},z)$ to present transmural distribution of myo- and collagen fibers orientation, respectively. Here, z denotes the normalized thickness varying between 0 at endocardium to 100 at epicardium along the transmural direction (denoted by x_3_), and $\theta^{m},\theta^{c}\in [-\pi /2,\pi /2]$ are used to denote the planar orientation of myo- and collagen fibers, respectively (Fig. 2).

Finally, it is important to clarify the distinction between the “large” (perymysial) collagen fibers that connect adjacent bundles of myofibers, and the “fine” (endomysial) collagen fibers that are woven around the individual myofibers and interconnect extensively with the thick collagen fibers (Fig. 12). At the image resolution used in our measurements (described earlier), the smaller endomysial collagen fibers that surround the myofibers are not discernable. Thus, we expect that the volume fraction $\phi^{c}$ and the fiber orientation distribution $\Gamma^{c}(\theta^{c},z)$ (presented in this work) predominantly represents perimysial fibers. The volume fraction of endomysial collagen fibers is shared between $\phi^{m}$ and $\phi^{c}$ (note that $\phi^{m}+\phi^{c}+\phi^{g}=1$).

### B. Constitutive model formulation

Recently, we developed a structural-based constitutive model for the tissue-level behavior of the RVFW myocardium. Here, we summarize the main considerations and assumptions underlying our model,^20^

(1) The fibrous phases, consisting of myofibers and undulated collagen fibers, are considered to be the mechanically dominant. The mechanical contribution of the non-fibrous ground substance with a small volume fraction $\phi^{g}$ was presented with a neo-Hookean term with a low stiffness $k^{g}$.
(2) The contributions of myofibers and large collagen fibers are directly accounted by incorporating detailed histologically measured information on their structure. In addition, the model accounts for the interaction between myo- and collagen fibers which is hypothesized to partly correspond to the presence of “fine” endomysial collagen fibers.
(3) We assume the affine kinematics for tissue constituents, i.e., all fibrous phases undergo the same deformation applied to the tissue.^23–25^ Also, noting that excised RVFW specimens, for both normal and PAH cases, were thin (thickness/width ratio of PAH specimens was about 0.11^14^), a plane-stress assumption was made in modeling the biaxial behavior of the specimens. More specifically, we modeled RVFW as a thin multi-layered material subjected to an in-plane strain-controlled loading. We assumed that there is no interaction between the layers and all the layers experience the same deformation and (approximately) produce a plane-stress state in each layer. This assumption was consistent with the histological measurements that the out-of-plane orientation of fibers is negligible. As a result, the out-of-plane components of stress were taken to be zero in the entire specimen subjected to biaxial tests while the in-plane stresses varied in the transmural direction (x_3_) due to the transmural variations in fiber orientation, fiber content, etc. The resultant stresses on the RVFW side faces are obtained by integrating the stress of all layers from endocardium to epicardium.

Following these considerations, we proposed the following strain energy function Ψ(C) for the RVFW myocardium:

$$\Psi (C)=\frac{\phi^{g}\;k^{g}}{2}(I_{1}-1)+\phi^{m}\;\Psi^{m}(I^{m})+\phi^{c}\;\left[\Psi^{c}(I^{c})+\Psi^{m-c}(I^{m},I^{c})\right],$$ (1)

where $C=F^{T}\;F$ is the right Cauchy-Green tensor with F being the deformation gradient tensor. Also, the kinematic invariants $I_{1}$, $I^{m}$, and $I^{c}$ in the above relation are defined as

$$I_{1}=tr(C),\;\;\;\;\;\;\;\;\;I^{m}=n^{m}\;\cdot \;C\;n^{m},\;\;\;\;\;\;\;\;\;I^{c}=n^{c}\;\cdot \;C\;n^{c},$$ (2)

where the unit vectors $n^{m}$ and $n^{c}$ characterize the local directions of myo- and collagen fiber ensembles. Also, $\Psi^{m}$ and $\Psi^{c}$ are the strain energy functions associated with myofibers and large (perimysial) collagen fibers, respectively, and $\Psi^{m-c}$ refers to the energy contribution of interaction between myo- and collagen fibers. At the tissue level, the second Piola-Kirchhoff stress tensor S is given as the sum of the following components:

$$S=\phi^{g}\;k^{g}(I-C_{33}C^{-1})+S^{m}+S^{c}+S^{m-c},$$ (3)

where I is the identity tensor, $S^{m}=2\;\phi^{m}\;\partial \Psi^{m}(C)/\partial C$, and $S^{i}=2\;\phi^{c}\;\partial \Psi^{i}(C)/\partial C$ (*i* = *c*, *m*-*c*), and the tissue is stress-free in the x_3_-direction.

Our experimental measurements of the content of myo- and collagen fibers in the RVFW specimens indicated that the variation of the collagen fiber volume fraction along the transmural direction was more pronounced in the post-PAH tissues than in normal tissues (Fig. 11). This observation suggested that, for the post-PAH tissues, the contribution of collagen fibers to the overall stress and, in turn, the amount of fiber-fiber interactions could have a significant transmural variation which needs to be addressed within our modeling framework. For this reason, we extended our model, previously developed for the normal RVFW,^20^ to include transmural variation in the volume fraction, collagen fiber recruitment properties, and the fiber-fiber interaction strength. The detail of this extension is described in this section.

#### 1. Myofibers

An exponential stiffening with strain was assumed for myofibers which, after being weighted by the 3-D orientation distribution function $\Gamma^{m}\left(\theta^{m},z\right)$, led to the following expression for the myofiber stress:

$$S^{m}=\frac{k_{1}^{m}}{H}\;\;\int_{0}^{H}\int_{-\pi /2}^{\pi /2}\phi^{m}(z)\;\Gamma^{m}\left(\theta^{m},z\right)\;\left(1-\frac{1}{\sqrt{I^{m}}}\right)\text{exp}\;\left[k_{2}^{m}{\left(\sqrt{I^{m}}-1\right)}^{2}\right]\;\;\left(n^{m}⊗n^{m}\right)\;\;d\theta^{m}\;dz\;,$$ (4)

for $I^{m}\geq 1$, and 0 otherwise, where $k_{1}^{m}>0$ is a stress-like material parameter, $k_{2}^{m}>0$ is a dimensionless parameter and *H* denotes the (normalized) tissue thickness.

#### 2. Collagen fibers

As previously discussed,^20,25^ we accounted for the undulation of collagen fibers such that a collagen fiber transmits load only if stretched beyond its slack stretch, denoted by $\lambda_{s}\geq 1$. Beyond this stretch, we assumed a linear force-displacement relation for the fiber. The resulting tissue-level stress for (large) collagen fibers is given by

$$S^{c}=\frac{k^{c}}{H}\;\;\int_{0}^{H}\int_{-\pi /2}^{\pi /2}\phi^{c}(z)\;\Gamma^{c}\left(\theta^{c},z\right)\;\left[\int_{\lambda_{lb}}^{\lambda_{ub}(z)}\frac{D(\lambda_{s},z)}{\lambda_{s}^{2}}\left(1-\frac{\lambda_{s}}{\sqrt{I^{c}}}\right)\;d\lambda_{s}\right]\;\left(n^{c}⊗n^{c}\right)\;d\theta^{c}\;dz,$$ (5)

where $k^{c}$ denotes the (tensile) modulus of a straight collagen fiber. In the above expression, $D(\lambda_{s},z)$ represents the distribution of undulation in an ensemble of collagen fibers with the slack stretch range $\lambda_{s}\in [\lambda_{lb},\lambda_{ub}(z)]$, where $\lambda_{lb}$ and $\lambda_{ub}$ denote the lower and upper bounds of collagen fiber ensemble recruitment stretch levels, with $\lambda_{ub}>\lambda_{lb}\geq 1$ and $\int_{\lambda_{lb}}^{\lambda_{ub}}D(\lambda_{s},z)\;\;d\lambda_{s}=1$. In our work, $D(\lambda_{s},z)$ is characterized by a scaled Beta distribution as

$$D(\lambda_{s},z)=\{\begin{array}{l} \frac{y^{\alpha -1}{\left(1-y\right)}^{\beta -1}}{B(\alpha ,\beta )\;[\lambda_{ub}(z)-\lambda_{lb}]},\;\mathrm{for}\;y\in \;[0,1]\\ 0,\;\;\;\;\;\;\;\;\;\text{otherwise} \end{array}\;,\;\;\;\;\;\;\;y=\frac{\lambda_{s}-\lambda_{lb}}{\lambda_{ub}(z)-\lambda_{lb}},$$ (6)

where B(α,β) is the Beta function with shape factors α and β. Note that $\lambda_{ub}$ is a function of the wall depth implying that the recruitment range of collagen fibers could vary transmurally. Our pilot studies of the tissue behavior consistently suggested that the collagen fibers in the midwall region are mechanically dominant whereas the collagen fiber closer to endo- and epicardial surfaces may not reach a full recruitment. Based on these observations, we assumed a simple exponential transmural variation for the upper bound stretch of the form

$$\lambda_{ub}(z)=\lambda_{ub}^{b}+(\lambda_{ub}^{m}-\lambda_{ub}^{b})\;\text{exp}\;[-k_{z}^{c}{\left(z-z^{c}\right)}^{2}/H],$$ (7)

where $k_{z}^{c}>0$, $\lambda_{ub}^{m}$, $\lambda_{ub}^{b}(>\lambda_{ub}^{m})$, and $0\leq z^{c}\leq 100$ are constants. In the above form, $\lambda_{ub}^{m}$ is the smallest value of $\lambda_{ub}$ that corresponds to the fastest recruitment with strain and takes place at $z=z^{c}$. For the values of $z^{c}$ close to the midwall ($40\leq z^{c}\leq 60$), $\lambda_{ub}^{b}$ is approximately the largest value of $\lambda_{ub}$ which corresponds to the slowest recruitment with strain and takes place at endocardial and epicardial surfaces (z=0,100). For later reference, the mean and standard deviation of the distribution (6), denoted by $\mu_{r}\in (\lambda_{lb},\lambda_{ub})$ and $\sigma_{r}$, respectively, are obtained as

$$\mu_{r}=(\lambda_{ub}-\lambda_{lb})\;\mu_{r}^{\prime }+(\lambda_{lb}-1),\;\;\;\;\;\;\;\sigma_{r}=(\lambda_{ub}-\lambda_{lb})\;\sigma_{r}^{\prime },$$

where

$$\mu_{r}^{\prime }=\alpha /(\alpha +\beta ),\;\;\;\;\;\;\;{\sigma^{\prime }}_{r}^{2}=\alpha \;\beta /[{\left(\alpha +\beta \right)}^{2}\;(\alpha +\beta +1)].$$ (8)

#### 3. Mechanical interactions

Finally, the stress term $S^{m-c}$ in (3) accounts for the coupling between myofibers and (large) collagen fibers using the combined invariant $\bar{I}=I^{m}+I^{c}$. The final from of this stress is expressed as

$$\begin{array}{c} S^{m-c}=\frac{1}{H}\;\;\int_{0}^{H}\;\int_{-\pi /2}^{\pi /2}\;\int_{-\pi /2}^{\pi /2}\phi^{c}(z)\;\Gamma^{m}\left(\theta^{m},z\right)\;\Gamma^{c}\left(\theta^{c},z\right)\;\;\{\psi \left(I^{m},I^{c},z\right)\;n^{m}⊗n^{m}\\ +\left\{\;\text{exp}\;\left[k_{2}^{mc}(\bar{I}-2)\right]-\text{exp}\;\left[k_{2}^{mc}(I^{m}-1)\right]\;\right\}\;\;n^{c}⊗n^{c}\}\;\;d\theta^{c}\;d\theta^{m}\;dz, \end{array}$$ (9)

where $k_{1}^{mc}>0$ and $k_{2}^{mc}>0$ are material parameters and

$$\psi \left(I^{m},I^{c},z\right)=k_{1}^{mc}(z)\left\{\;\text{exp}\;\left[k_{2}^{mc}\left(\bar{I}-2\right)\right]-\left[k_{2}^{mc}\left(I^{c}-1\right)+1\right]\text{exp}\;\left[k_{2}^{mc}(I^{m}-1)\right]\right\}.$$ (10)

Following our finding mentioned in the context of relation (7), a regionally earlier and faster recruitment of collagen fibers with strain suggests a stronger and weaker interaction with myofibers, respectively. Therefore, consistent with the form (7), we assumed the following exponential (transmural) variation for the stiffness-like constant $k_{1}^{mc}(z)$:

$$k_{1}^{mc}(z)=k_{1b}^{mc}+(k_{1m}^{mc}-k_{1b}^{mc})\;\text{exp}\;[-k_{z}^{mc}{\left(z-z^{mc}\right)}^{2}/H],$$ (11)

where $k_{z}^{mc}>0$, $k_{1b}^{mc}>0$, $k_{1m}^{mc}>k_{1b}^{mc}$, and $0\leq z^{mc}\leq 100$ are constants. More specifically, $k_{1m}^{mc}$ is the largest value of $k_{1}^{mc}$ that corresponds to the highest interaction and takes place at $z=z^{mc}$. For the values of $z^{mc}$ close to the midwall ($40\leq z^{c}\leq 60$), $k_{1b}^{mc}$ is approximately the smallest value of $k_{1}^{mc}$ which corresponds to the lowest interaction and occurs at endocardial and epicardial surfaces (*z* = 0,100). Finally, we note that, the expression (9) is a reduced version of the original expression in our previous work,^20^ where the recruitment was included in the interaction term. The simplified form (9) is computationally tractable and still very useful to estimate the interaction contribution.

### C. Parameter estimation

Each RVFW specimen was tested for five strain protocols: $E_{11}=\alpha \;E_{22}$, α=0.25, 0.5, 1, 2, 4, where E=(C−I)/2 is the tissue-level Green-Lagrange strain tensor. The experimental data were processed to obtain $S_{11}-E_{11}$ and $S_{22}-E_{22}$ plots. A plane stress state (*S*_33_ = *S*_13_ = *S*_23_ = 0) was assumed. Given the collected data on mechanical testing, the continuous transmural distributions of myo- and collagen fibers (interpolated from histological data), the data on the volume content (Fig. 11), and the constitutive relation (3) for the tissue-level behavior of the RVFW, best-fit parameters were estimated using a non-linear least square minimization with the objective function

$$\Sigma (\delta )=\sum_{i=1}^{N}{\left[{\left(S_{11}(\delta )-{\hat{S}}_{11}\right)}^{2}+{\left(S_{22}(\delta )-{\hat{S}}_{22}\right)}^{2}\right]}_{i},$$ (12)

where $\delta_{j}$ denotes the set of unknown parameters, N is the total number of data points, and ${\hat{S}}_{ij}$ denotes the stress values obtained from the experimental biaxial tests. A few remarks with regard to the estimation of the parameters are in order

(1) The similar sequential procedure, as previously described,^20^ was used to estimate the parameters. Briefly, the lower bound strain $E_{lb}=(\lambda_{lb}^{2}-1)/2$ and myofiber material parameter $k_{1}^{m}$ were directly estimated from the mechanical data in the low strain regime based on the hypothesis that the behavior of the tissue is governed by the response of the myofibers in the low strain regime as no collagen fiber is recruited in this regime. The remaining unknown parameters were estimated using the multi-protocol strain-stress data for the entire strain range. The quality of fit of fully estimated model for low strain regime was minimally different from the (original) fit of myofiber response ($k_{1}^{m}$) to this regime.
(2) In order to present the results as functions of strain, the unknown parameters $\lambda_{lb}$, $\lambda_{ub}^{b}$, and $\lambda_{ub}^{m}$ were properly replaced in terms of $E_{lb}$, $E_{ub}^{b}=[{\left(\lambda_{ub}^{b}\right)}^{2}-1]/2$, and $E_{ub}^{m}=[{\left(\lambda_{ub}^{m}\right)}^{2}-1]/2$, respectively. Correspondingly, the reported values of $\mu_{r}$ and $\sigma_{r}$ reflect the values in the strain space.
(3) Our pilot studies to evaluate different forms of a transmural recruitment function consistently indicated that, for the case of post-PAH tissues, there are sections of the RVFW at which collagen fibers do not reach a full recruitment and their interaction with myofibers is minimal. Based on these considerations, we chose the values $E_{ub}^{b}=0.4$ and $k_{1b}^{mc}=0.01$(kPa) *a priori* corresponding to the sections in the RVFW with very slow fiber recruitment and negligible interaction.
(4) The mechanical contribution of the amorphous matrix was taken to be small (compared to those of myo- and collagen fibers), and the value $k^{g}=10\;\mathrm{kPa}$ was assigned *a priori*.

### D. Post-estimation evaluations

#### 1. Significance of transmural variations

The importance of the terms representing the tranmural varations in collegen fiber recuritment and the strengh of the interaction [introduced in Eqs. (7) and (11)] was assessed using Akaike Information Criterion (AIC), defined as

$$\mathrm{AIC}=N\;\;\ln\left(\frac{1}{N}\Sigma (\delta )\right)\mathrm{+2}\;\mathrm{K,}$$ (13)

where *K* is the number of parameters, i.e., length of δ. The AIC were calculated before and after incorporating the parameters $\kappa =\{k_{z}^{c},z^{c},k_{z}^{mc},z^{mc}\}$ in the model.

#### 2. Parameter correlations

Next, the determinability of the parameters was evaluated using the calculations of the correlation matrix. As described in the previous study,^20^ assuming zero-mean, uncorrelated errors, the coefficient $CR_{\mathrm{pq}}$, defining the correlation between the parameters $\delta_{p}$ and $\delta_{q}$ is approximately given by^26^

$$CR_{\mathrm{pq}}\approx \frac{{\left(H^{\mathrm{-1}}\right)}_{\mathrm{pq}}}{\sqrt{{\left(H^{\mathrm{-1}}\right)}_{\mathrm{pq}}}\sqrt{{\left(H^{\mathrm{-1}}\right)}_{\mathrm{pq}}}},\;\mathrm{(no}\;\;\mathrm{sum}\;\mathrm{on}\;p\;\;\text{and}\;\;q).$$ (14)

where $H=2\;J^{T}J$ and **J** is the sensitivity matrix defined as the derivative of the descriptor function in Eq. (12) [i.e., $S_{ij}(\delta )$] with respect to the unknown parameters δ calculated at the optimal values $\delta^{*}$. Note that H is the approximate Hessain matrix for the oridinary least-square optimization when the descriptor $S_{ij}(\delta )$ is replaced by its first-order Taylor expansion.^27^

### E. Wall stress estimation

The restoration of myocardial wall stress is known to be a key underlying feedback mechanism to control rate and extent of growth and remodeling of the RVFW during PAH.^5^ The RVFW is a heterogeneous, nonlinear and anisotropic biological tissue, and accurate calculation of transmural distribution of wall stress will require a detailed, 3D finite element model of the heart ventricles with the implementation of proper material models. However, for gaining a preliminary understanding of the relationships between the wall stress and the remodeling of the RVFW under PAH, an approximate equilibrium model of a simplified geometry of the heart ventricles may be used. Such models, also known as Laplace-type models, have been frequently used to estimate the average wall stress components in the LV by assuming a simple prolate spheroidal geometry for the LV chamber. In this work, to estimate the RV wall stress, we used a simplified truncated heart model to approximate the biventricular shape up to the equatorial plane [see Figs. 10(a) and 10(b)]. This simplified geometry has been frequently used for heart simulations,^28,29^ and it assumes a prolate spheroidal shape for the left ventricle overlapping a general ellipsoidal shape to form the right ventricle. The model provides a fair approximation for the biventricular shape in the rat hearts [see Fig. 1(a)]. Using this geometry, we conducted an approximate equilibrium analysis to estimate RV wall stress explained as follows.

The only load on the RVFW was assumed to be the internal pressure *p* in RV. We considered an element cut from the central region of the RVFW [see Fig. 10(c)] to calculate the *average* wall stress at the equator. Using a Laplace law-like approach^30,31^ and ignoring shear forces and bending moments, a force balance in the radial direction on this element leads to

$$p\;r_{C}\;r_{L}\;d\varphi_{C}\;d\varphi_{\;L}=2\;dF_{\text{CC}}\;\text{sin}\left(\frac{d\varphi_{C}}{2}\right)+\;dF_{\text{LL}}\;\text{sin}\left(d\varphi_{\;L}\right)\approx dF_{\text{CC}}\;d\varphi_{C}+dF_{\text{LL}}\;d\varphi_{\;L},$$ (15)

where $r_{C}$ and $r_{L}$ are the radii of curvature at the RV inner wall associated with the angles $d\varphi_{C}$ and $d\varphi_{\;L}$. respectively [Fig. 10(c)], and the forces $dF_{\text{CC}}$ and $dF_{\text{LL}}$ are the total forces acting along the circumferential and longitudinal directions, respectively [Fig. 10(c)]. The total forces are related to the corresponding average wall stress through the relations

$$dF_{\text{CC}}\;=\sigma_{\text{CC}}\;dA_{C},\;dF_{\text{LL}}\;=\sigma_{\text{LL}}\;dA_{L},$$ (16)

where $\sigma_{\text{CC}}$ and $\sigma_{\text{LL}}$ are the average circumferential and longitudinal Cauchy stress components acting on the cut element from the RVFW [Fig. 10(c)]. Also, in the above relations, $dA_{C}$ and $dA_{L}$ are the areas associated with the circumferential and longitudinal forces, respectively [Fig. 10(c)], and given by

$$dA_{C}=\;(R_{L}^{2}-r_{L}^{2})\;\frac{d\varphi_{\;L}}{2\;},\;dA_{L}=\;(R_{C}^{2}-r_{C}^{2})\;\frac{d\varphi_{C}}{2\;},$$ (17)

where $R_{C}$ and $R_{L}$ are the radii of curvature at the RV outer wall. Replacing relations (16) together with relations (17) into (15), we arrive at

$$p=\frac{1}{2\;r_{C}\;r_{L}}\left[\sigma_{\text{CC}}\;(R_{L}^{2}-r_{L}^{2})+\sigma_{\text{LL}}\;(R_{C}^{2}-r_{C}^{2})\right].$$ (18)

An additional equation accompanying the above equation is the overall force balance on the equatorial plane [Fig. 10(b)] in the longitudinal direction given by

$$p\;A_{1}=\sigma_{\text{LL}}\;A_{2},$$ (19)

where

$$A_{1}=\frac{\pi \;}{2}[(a-w)\;(b-w)-b^{2}]+\Delta ,\;A_{2}=\frac{\pi \;}{2}[\text{ab}-b^{2}]-A_{1},$$

with

$$\Delta =t\;b^{2}-(a-w)\;(b-w)\;{\text{tan}}^{-1}\left(\frac{b-w}{a-w}\text{tan}(t)\right),\;t={\text{tan}}^{-1}\left(\frac{b-w}{a-w}\sqrt{\frac{{\left(a-w\right)}^{2}-b^{2}}{2\text{wb}-w^{2}}}\right).$$

Note that Eq. (18) assumes a uniform $\sigma_{\text{LL}}$ on the equatorial plane in the RVFW. The radii of curvatures in Eq. (18), calculated for the cut element, are obtained as

$$R_{C}=\frac{b^{2}}{a},\;r_{C}=\frac{{\left(b-w\right)}^{2}}{a-w},\;R_{L}=a,\;r_{L}=a-w.$$ (20)

The above relations are based on the assumption that the RV ellipsoid in our biventricular model [i.e., the intercepted ellipsoid in the left in Fig. 10(a)] is an oblate spheroid with the semi-axes (*a,a,b*). Such a simplifying assumption was needed because our measurements of the RV dimensions were not sufficient to completely quantify the dimensions of a general ellipsoidal shape model for the right heart in the Laplace model [Fig. 10(a)]. Finally, substituting the above relations into Eqs. (18) and (19), the stress components $\sigma_{\text{CC}}$ and $\sigma_{\text{LL}}$ are expressed in terms of the RV cavity pressure p and geometrical parameters a, b, and w.

Our morphological study performed on normal (n = 3) and hypertensive (n = 3) murine excised hearts did not include direct measurement of the parameters a and b; however, it provided us with the measurements of the thickness of the RVFW, the circumferential and longitudinal lengths of the RVFW and the volume of the RV cavity. We estimated the unknown parameters a and b from these measurements using geometrical relationships within the simplified model in Figs. 10(a) and 10(b). Details of this analysis are given in supplementary material. Finally, we note that we use the equilibrium equations (18) and (19), derived based on the Laplace model, to provide insights into the qualitative contrast in the regulatory role of circumferential and longitudinal components of *in-vivo* wall stress on the structural and mechanical adaptations of the RVFW. In fact, these equations can provide preliminary predications for the components of an average wall stress at end-systole without the knowledge on the active material model or internal structure. However, we emphasize that the accurate prediction of the local wall stress will require developing a complete biventricular finite-element model with appropriate material laws for passive and active behaviors of myocardium.

## VI. SUPPLEMENTARY MATERIAL

See supplementary material for the procedure to estimate the parameters *a* and *b* using the measurements of the RVFW dimensions and the RV volume. These parameters were needed to compute the wall stress components in Eqs. (18) and (19).
